# Mechanical signaling regulates vascular smooth muscle cell adaptation in aging

**DOI:** 10.3389/fphys.2025.1593886

**Published:** 2025-07-14

**Authors:** Amin Mohajeri, Song Yi Shin, Samuel Padgham, Devon J. Boland, Dana Pittman Ratterree, Jacob Blizman, Gang Han, Christopher R. Woodman, Andreea Trache

**Affiliations:** ^1^ Department of Kinesiology and Sport Management, Texas A&M University, College Station, TX, United States; ^2^ Department of Medical Physiology, Texas A&M University Health Science Center, Bryan, TX, United States; ^3^ Texas A&M Institute for Genome Sciences & Society, Texas A&M University, College Station, TX, United States; ^4^ Department of Epidemiology and Statistics, Texas A&M University School of Public Health, College Station, TX, United States; ^5^ Department of Biomedical Engineering, Texas A&M University, College Station, TX, United States

**Keywords:** aging, integrins, actin, mechanical stimulation, vascular smooth muscle

## Abstract

Aging is an independent risk factor for cardiovascular disease. Preventing age-induced arterial dysfunction and the associated risk of cardiovascular disease remains a significant clinical challenge. Aerobic exercise, which induces a temporary increase in both blood flow and pressure in active tissue, has been shown to reduce macroscale arterial stiffening in humans. This study investigates the effects of mechanical stimuli on improving aging pathophysiology of vascular smooth muscle (VSM) cells isolated from soleus feed arteries (SFA). We hypothesized that age-induced impairment of VSM contractility can be rescued by mechanical stimulation that enhances formation of smooth muscle alpha-actin (SMα-actin) fibers and cell-matrix adhesions in aged VSM cells. *Ex-vivo* functional studies were used to assess myogenic contractility of VSM in isolated SFA from young (4 months) and old (24 months) Fischer 344 rats. These data indicated that pre-treatment of isolated aged SFA with a short-duration increase in intraluminal pressure rescued contractility. The mechanical stretch-induced remodeling of the cellular architecture was assessed in VSM cells isolated from young and old SFA. To dissect the mechanisms involved, the structural and functional properties of VSM cells were assessed by using mechanical stimulation combined with fluorescence confocal microscopy. Results showed that aged VSM cells respond faster than young cells to 2D biaxial cyclic stretch by increasing actin stress fiber formation and vinculin recruitment at cell-matrix adhesions. In addition, hydrostatic pressure treatment applied to aged VSM cells plated on stiffer substrates restored actin fibers and integrin β1 recruitment. Taken together, these findings suggest that discrete VSM cell mechanical properties and their ability to adapt to external mechanical signals are key in restoring VSM contractility in aging. These results are significant because they provide a novel understanding of the mechanisms by which mechanical stimulation improves VSM contractility in aged resistance arteries. Our results provide new insights into the role of VSM in vascular aging and highlight a new direction for mitigating age-related effects via mechanical stimulation-induced VSM remodeling.

## 1 Introduction

Increased lifespan (Gulland, 2016) leads to an increase in cardiovascular risks in elderly, that can be associated with an age-related decline in vascular function (Strait and Lakatta, 2012; James et al., 2006). Arteries stiffen with age (Sehgel et al., 2015), thereby reducing their ability to dampen the blood pressure wave as it travels to the microcirculation (Mitchell et al., 2004; O'Rourke and Safar, 2005). Smaller arteries contain comparable amounts of vascular smooth muscle (VSM) cells and extracellular matrix (ECM) (Dinardo et al., 2014). Thus, VSM cells play a crucial role in the regulation of vascular function in resistance arteries, while the matrix represents the passive component of the vascular wall that VSM cells work on.

VSM cells regulate vascular tone and blood flow in response to intraluminal pressure changes. It has been shown that contractile function of VSM cells in resistance arteries is affected by aging (Seawright et al., 2018; Seawright et al., 2016). This suggests that aged VSM cells experience a phenotypic shift towards a more synthetic and hypertrophic state, eventually leading to elevated wall stiffness and reduced contractile capacity (Muller-Delp et al., 2018). Other recent studies have shown that contractility of VSM cells is also determined by their cellular architecture, shape and organization (Alford et al., 2011). Variations in cell shape can significantly affect the contractile behavior of VSM cells in an *in vitro* reconstituted tissue, indicating that structural organization at cellular and tissue levels may contribute to augmenting their functional contractile properties. Besides regulating arterial contractility in response to hemodynamic changes, VSM cells are also responsible for regulating ECM deposition, and its organization via interactions between the actomyosin contractile unit and cell adhesions that connect the cell to extracellular matrix (Saphirstein et al., 2013; Lakatta, 2003).

Extracellular matrix provides both structural support and means for transmission of biochemical signals needed for the regulation of VSM cellular function. The stiffness of the ECM has been identified as a mechanoregulator of VSM cellular function (Steucke et al., 2015). Integrins are transmembrane receptors that anchor the cells within the extracellular matrix via cell-matrix adhesion proteins (Harburger and Calderwood, 2009). Due to their spatial positioning in the cell, integrins function as mechanoreceptors sensing forces from the extracellular matrix as well as intracellular actomyosin contractility state (DeMali et al., 2003). Their expression and function have been shown to influence VSM survival and vasoregulation (Turlo et al., 2013). While VSM cells express several integrins (Moiseeva, 2001), the RGD (Arg-Gly-Asp) binding integrins α5β1 and αvβ3 have important and distinct roles in regulating vascular function with both integrins being activated to regulate vascular contractility in response to increased pressure (Martinez-Lemus et al., 2005). Disruption of these processes can lead to alteration in contractility and, consequently, to vascular dysfunction.

Aerobic exercise reduces macroscale arterial stiffening in elderly (Tanaka et al., 2000; Seals et al., 2008; Santos-Parker et al., 2014). During exercise, both blood flow and pressure increase in the active tissue. It is well accepted that the vascular benefits of exercise are mediated, in part, by increased shear stress on the endothelium (Herrera et al., 2010). However, short-duration increases in arterial pressure induced by exercise (Overton et al., 1988) also improve vascular contractility in resistance arteries in an age-dependent manner (Ghosh et al., 2015; Ojha et al., 2022; Seawright et al., 2016). Therefore, exercise may mitigate the effects of aging on vascular contractility by two distinct mechanisms. First, exercise-induced shear stress improves endothelial function and restores nitric oxide production that has been well studied (Taddei et al., 2000; Laughlin et al., 2008; Trott et al., 2009; Luttrell et al., 2013). Second, repeated short-duration wall stretch (Lu and Kassab, 2011), due to aerobic exercise-induced arterial pressure elevation (Overton et al., 1988), generates long-term changes in VSM phenotype restoring normal VSM contractile function (Ghosh et al., 2015; Muller-Delp et al., 2018). However, molecular mechanisms by which exercise restores arterial compliance and improves VSM contractile responses in aged resistance arteries (Donato et al., 2007) are not fully understood.

The mechanical stretch of the vessel wall induced by the intraluminal pressure increases during exercise, activates the mechanosensitive signaling cascade which enhances expression of contractile proteins and therefore the overall contractile phenotype of aged VSM cells can be improved. Mechanical stretch of the arterial wall due to the increase of intraluminal pressure from 50–110 cm H_2_O alters conformation of β-integrins (Katsumi et al., 2005; El-Yazbi and Abd-Elrahman, 2017) and triggers assembly of cell-matrix adhesions (Alenghat and Ingber, 2002; Geiger and Bershadsky, 2001). This is followed by RhoA/ROCK-induced actomyosin activation, which is necessary to redistribute physical forces needed for cell contraction and to enable cell adaptation to the extracellular microenvironment (Chen et al., 2013; Zhang and Gunst, 2006). Further elevation of intraluminal pressure above 110 cm H_2_O triggers formation of actin stress fibers (Flavahan et al., 2005) presumably through enhanced actin polymerization (Cipolla et al., 2002). In turn, this stimulates myocardin-related transcription factor-A translocation to the nucleus where it interacts with serum response factor enhancing transcription of contractile proteins (Lacolley et al., 2017). In addition, an increase in the intraluminal pressure may lead to an elevation of myosin light chain phosphorylation, thus, further enhancing VSM cell contractility (Cole and Welsh, 2011).

This study investigates the effects of exercise-like *in vitro* mechanical stimuli on improving aging pathophysiology of VSM cells isolated from soleus feed arteries (SFA). Our study focuses on SFA because they have an integral role in regulating blood flow to the soleus muscle at rest and during exercise, and their myogenic tone contributes to control of peripheral resistance and blood flow (Ghosh et al., 2015; Donato et al., 2007; Williams and Segal, 1993). Thus, we aimed to study how mechanical stimulation induces remodeling of the aged VSM cell architecture. We hypothesized that age-induced impairment of VSM contractility can be rescued by mechanical stimulation that enhances formation of smooth muscle alpha-actin (SMα-actin) fibers and cell-matrix adhesions in aged VSM cells. The hypothesis was tested by using *ex-vivo* functional experiments in cannulated SFA, and high-resolution confocal microscopy combined with mechanical stimulation experiments of VSM cells.

## 2 Methods

### 2.1 Animals

Young (4 months) and old (24 months) male Fischer 344 rats were obtained from the National Institute on Aging (NIA) and housed at the Texas A&M Comparative Medicine Program Facility. The animals were kept under a 12:12 h light-dark cycle and provided food and water *ad libitum*. The study was approved by Texas A&M University Institutional Animal Care and Use Committee. In agreement with the current institutional regulations, the animals were examined daily by Animal Care Facility veterinarians and by study investigators.

### 2.2 Isolation of soleus muscle feed arteries

To isolate SFA from young and old Fischer 344 rats, similar protocols were applied as mentioned in our previous works (Seawright et al., 2018; Trott et al., 2009). Briefly, a cocktail of Xylazine (5 mg/kg body weight, 033197, Covetrus, TX) and Ketamine (80 mg/kg body weight, 071069, Covetrus, TX) was injected intraperitoneally. Once the animal was anesthetized, the soleus-gastrocnemius-plantaris muscle complex was dissected from each hindlimb and placed in a physiological saline solution (PSS) buffered with MOPS (pH 7.4), and maintained at a temperature of 4°C. The PSS/MOPS buffer contained: 145 mM NaCl, 4.7 KCL, 2 mM CaCl2, 1.17 mM MgSO4, 1.2 mM NaH2PO4, 5 mM glucose, 2 mM pyruvate, 0.02 mM EDTA, and 25 mM MOPS. A Lucite chamber filled with MOPS-PSS at 4°C was used to preserve the dissected SFA throughout the cannulation procedure. Rats were euthanized by excising the heart. Except otherwise noted, all reagents were purchased from Sigma (St. Louis, MO).

### 2.3 Cannulation of arteries and assessment of myogenic constriction response

SFA were cannulated with two glass micropipettes and secured with surgical thread. The micropipettes were subsequently attached to separate hydrostatic pressure reservoirs filled with MOPS-PSS supplemented with albumin (1 g/100 ml, 10856, Affymetrix, CA). SFA were pressurized to 60 cm H_2_O (1 mm Hg = 1.36 cm H_2_O) and checked for leaks. When a SFA was determined to be leak free, intraluminal pressure was raised to 90 cm H_2_O (p90) or 130 cm H_2_O (p130) for 60 min. Intraluminal pressures of 90 and 130 cm H_2_O were used to match the pressures believed to be present in rat SFA at rest and during exercise, respectively (Williams and Segal, 1993). At the end of the 60 min pressure treatment period, intraluminal pressure in the p130 SFA was lowered to 90 cm H_2_O, and SFA were allowed to develop stable tone. After achieving stable tone for 10 min, myogenic responses were assessed using step-increases in pressure up to 135 cm H_2_O in 15 cm H_2_O increments followed by step-decreases down to 45 cm H_2_O. At the end of experiment, cannulated SFA were incubated in Ca^2+^-free PSS for 30 min to obtain the maximal passive diameter. Thus, myogenic response data were expressed as percent constriction [(Dmax–Dc)/Dmax] × 100, where Dc is the measured diameter for a given pressure, and Dmax is maximal passive diameter measured in Ca^2+^-free PSS (Ghosh et al., 2015).

### 2.4 Vascular smooth muscle cell culture

VSM cells were isolated from the SFA dissected from young and old Fischer 344 rats using similar techniques as described in our previous publication (Seawright et al., 2018). VSM cells were grown in Dulbecco’s Modified Eagle Medium F-12 supplemented with 10% fetal bovine serum and 10 mM HEPES (Sigma, St. Louis, MO), 2 mM L-glutamine, 1 mM sodium pyruvate, 100 U/ml penicillin, 100 μg/ml streptomycin and 0.25 μg/ml amphotericin B and set in an incubator at 37°C and 5% CO_2_. Except otherwise noted, all reagents were purchased from Invitrogen (Carlsbad, CA).

### 2.5 RNA-sequencing

#### 2.5.1 RNA extraction, library preparation and processing

Total RNA was extracted from cultured VSM cells isolated from both young and old SFA using TRIzol (15596026, Thermo Fisher, Waltham, MA) according to the manufacturer’s protocol. RNA concentration was determined using a Qubit fluorometer (Thermo Fisher, Waltham, MA), and RNA integrity was assessed with an Agilent TapeStation using RNA ScreenTape (5,067–5,579, Agilent, Lexington, MA). Samples with sufficient quality were normalized to a final concentration of 20 ng/µL for library preparation. Stranded mRNA sequencing libraries were prepared using the Illumina TruSeq Stranded mRNA Library Preparation Kit (20020594, Illumina, San Diego, CA) following the manufacturer’s instructions. Libraries were then quantified, normalized, and pooled at equimolar concentrations for sequencing. The RNA libraries were sequenced on the NovaSeq 6,000 (Illumina, San Diego, CA) platform in a 2x100bp approach. Total sequencing depth ranged from 26.9M–33.1M reads per sample. Raw sequencing files were base called and converted into FASTQ files using the DRAGEN BCL Convert application (Illumina, San Diego, CA).

All RNA-sequencing files were processed identically using a series of bioinformatics tools organized and executed within the Snakemake workflow manager (Köster and Rahmann, 2012). Briefly, FASTQ files were trimmed of any adapter, barcode, or low quality (Phred <20) sequenced using Cutadapt (v3.5) (Martin, 2011) and FastQC (v0.11.9) with the TrimGalore (v0.6.7) wrapper. MultiQC (v1.27.1) was used to compile FASTQ quality metrics pre- and post-trimming to visually confirm the removal of non-biologically relevant and low-quality scoring bases that could impact mapping and quantification steps (Ewels et al., 2016). Trimmed reads were then mapped to the *R. norvegicus* (v6.0) reference genome assembly (RefSeq: GCF_000001895.5) using the STAR (v2.7.10b) aligner (Dobin et al., 2013). Gene-level counts corresponding to annotated gene models from the *R. norvegicus* GTF (RefSeq: GCF_000001895.5) file were extracted from the STAR-aligned BAM files using featureCounts form the Subread (v2.0.8) package (Liao et al., 2019). Gene-level count tables were used for downstream statistical testing.

#### 2.5.2 Differential gene expression (DGE) analysis

DESeq2 (v1.46.0) was used to identify differentially expressed genes between the old and young age groups (Love et al., 2014). First gene-level count tables for each sample were combined into a single count matrix in R. A dds object was created with the gene-level count matrix and the model design “∼Age”. The young samples were leveled as the reference for all downstream hypothesis testing. To focus DGE testing on genes with a moderate level of detectable expression in at least one of the two age groups, low count genes were filtered from the dds object. These low count level genes were removed if they had a total count of less than 10 in at least three of the six samples tested. After standardization and model fitting, differential expression testing was conducted using the Wald’s test. DEGs were identified as those displaying a Benjamini-Hochberg (BH) corrected p-value (p-adj) < 0.05 and a |log_2_ Fold change| ≥ 1. Volcano plots and heatmaps were rendered using the EnhancedVolcano (v1.24.0) (Blighe et al., 2024) and ComplexHeamtap (v2.22.0) (Gu et al., 2016) R packages, respectively.

#### 2.5.3 Pathway enrichment analysis

DOSE (v4.0.0) (Yu et al., 2014), clusterProfiler (v4.14.4) (Wu et al., 2021), and org.Rn.eg.db (v3.20.0) R packages were used to perform annotated pathway enrichment from the DEGs identified in the RNA-sequencing data. Both Gene Ontology (GO) (The Gene Ontology Consortium et al., 2023) and Kyoto Encyclopedia of Genes and Genomes (KEGG) (Kanehisa et al., 2010; Kanehisa et al., 2017) databases were queried for pathway enrichment. GO terminology were tested for significant pathway enrichment with the “enrichGO” function of clusterProfiler, with the org.Rn.eg.db package. KEGG terms were tested for significant enrichment by querying the DEGs against the *R. norvegicus* (organism = “rno”) models, using the “enrichKEGG” function of clusterProfiler. Significantly enriched terms were identified as having a Benjamini-Hochberg (BH) corrected p-value (p-adj) < 0.05 and a fold enrichment of greater than 0.

### 2.6 Vascular smooth muscle cells mechanical stimulation *in vitro*

#### 2.6.1 Cyclic stretch

To determine the effect of the mechanical stretch on VSM cells morphology *in vitro*, we used a custom-made cyclic equibiaxial stretch device (Na et al., 2008). The components of the custom-made device were sterilized in an ethanol bath followed by 20 min UV exposure. Following sterilization, the edge of the frame was lightly coated with sterile vacuum grease and the silicon membrane (70P001-200-005, SMI, Saginaw, MI) was set over the frame and then the membrane was locked in place with a fitted ring. Upon the completion of these steps, the membranes were coated with fibronectin and then incubated at 37°C for 3 h. VSM cells were cultured on fibronectin-functionalized membranes for 24 h and then exposed to 10% equibiaxial cyclic stretch at 0.25 Hz (Na et al., 2008) for different time durations 2, 5, 15, and 30 min. No stretch, static condition was used as control. At the end of the experiment, cells were fixed in their stretched state and then immunofluorescently labeled for the proteins of interest.

#### 2.6.2 Pressure treatment

To investigate the effect of pressure increases on the VSM cells *in vitro*, we used a pressure stimulator MechanoCulture TR Hydro mechanical stimulation system (CellScale, Waterloo, Canada). VSM cells were cultured on hydrogels of different stiffnesses mimicking young and old vessel wall matrix stiffness at 4 kPa (ss12-EC-4-EA) and 100 kPa (SS12-EC-100-EA), respectively (Matrigen, Irvine, CA). After 24 h the coverlips with cell cultures were transferred in the specimen chamber of the MechanoCulture TR pressure stimulator, which was subsequently filled with cell culture medium and sealed. The chamber was connected to an air compressor which was programmed to expose the cells to an *in vitro* static pressure of 16 kPa (∼160 cm H_2_O) for 30 min (Swiatlowska et al., 2022). At the end of the stimulation protocol, coverslips were transferred to a petri dish for immediate fixation and immunofluorescence labeling.

### 2.7 Assessment of vascular smooth muscle cell morphology

#### 2.7.1 Immunofluorescence labeling

Upon the completion of mechanical stimulation experiments, VSM cells were fixed in 2% paraformaldehyde (Electron Microscopy Sciences; Hatfield, PA) in DPBS for 10 min. After washing with a glycine buffer, VSM cells were incubated overnight at 4°C with specific primary antibodies mouse anti-smooth muscle α-actin IgG2a (SMα-actin, A5228), mouse anti-vinculin (MAB3574) (St. Louis, MO), hamster anti-integrin-β1 conjugated with Alexa 488 (102211, Biolegends, San Diego, CA), or rabbit anti-phospho-cofilin (p-cofilin, 3311S) (Cell Signaling Technology, Danvers, MA) in a sodium citrate buffer containing 1% BSA and 0.05% Triton-X (Sun et al., 2005; Seawright et al., 2018). Cells were then washed and incubated at room temperature for 1 h with goat anti-mouse Rhodamine Red-X (115–295–206), donkey anti-rabbit Alexa 647 (711–605–152), or goat anti-mouse Alexa 488 (115–545–205) (Jackson Immunoresearch, West Grove, PA) followed by another round of washing, and then immediate imaging in DPBS.

#### 2.7.2 Vascular smooth muscle cell imaging

Cell imaging experiments were performed on a confocal microscope Olympus Fluoview FV3000 system equipped with a UPLSAPO 40XS silicon oil 1.25 NA objective lens (stretch experiments) and a UPLSAPO 20X 0.75 NA (pressure treatment experiments). Fluorescence images of VSM cells were captured as 3D image stacks of 20–30 planes at 0.5 μm step size, which were presented as xy projections.

#### 2.7.3 Fluorescence image analysis

To quantify the stretch induced alterations of specific proteins, measurements of the cell area and specific protein area were performed by using the masking tool and image statistics tools in the SlideBook 6 software (Intelligent Imaging Innovations, Denver, CO) (Lim et al., 2010). For each cell, protein area was determined based on fluorescence intensity threshold masking for specific cell structures (e.g., fibers or focal adhesions) and was further normalized to cell area.

To quantify pressure-induced changes of protein levels by immunofluorescence, individual cells were masked using Labkit plugin (Arzt et al., 2022) for ImageJ/Fiji (Schindelin et al., 2012). A visual comparison between the automatically generated masks and original images was performed and overlapping cell masks were manually separated. Mean fluorescence intensity for each protein (SMα-actin, vinculin, phospho-cofilin) was then determined within the mask of each cell by measuring total fluorescence intensity and normalizing to the respective cell area.

### 2.8 Statistical analysis

Myogenic response curves were analyzed by two-way repeated measures ANOVA to detect differences between (young vs. old). Alpha level ≤0.05 was the threshold for statistical significance defined as p < 0.05. All data are presented as mean ± SE.

Imaging experiments were conducted on VSM cells isolated from SFA from Fischer 344 rats (n = 2-3 animals/condition). For each cell, the fluorescence protein area was normalized to the cell area as specified above to enable comparison of a larger number of cells for statistical analysis. The normality assumption was checked using Q-Q plot and Shapiro-Wilk test prior to conducting one way ANOVA. Alpha level less or equal to 0.05 was the threshold for statistical significance defined as p-value <0.05. STATA software v. 17.0 BE (Stata LLC, College Station, TX) was used to conduct the analysis.

## 3 Results

### 3.1 Characteristics of rats and SFA

The age of young rats was 4 months (n = 5), and the age of old rats was 24 months (n = 4). The body weights for old rats (442 ± 6 g) were significantly higher than those of young rats (337 ± 14 g). The maximal passive diameters were not significantly different between groups (young 184.6 ± 18.9 µm vs. old 190.4 ± 28.1 µm). Differences in animal weight and maximal diameters of arteries between groups (young vs. old) were analyzed using t-tests and one-way ANOVA among groups.

### 3.2 RNA-sequencing displays an age-dependent pattern of gene expression

**FIGURE 1:**
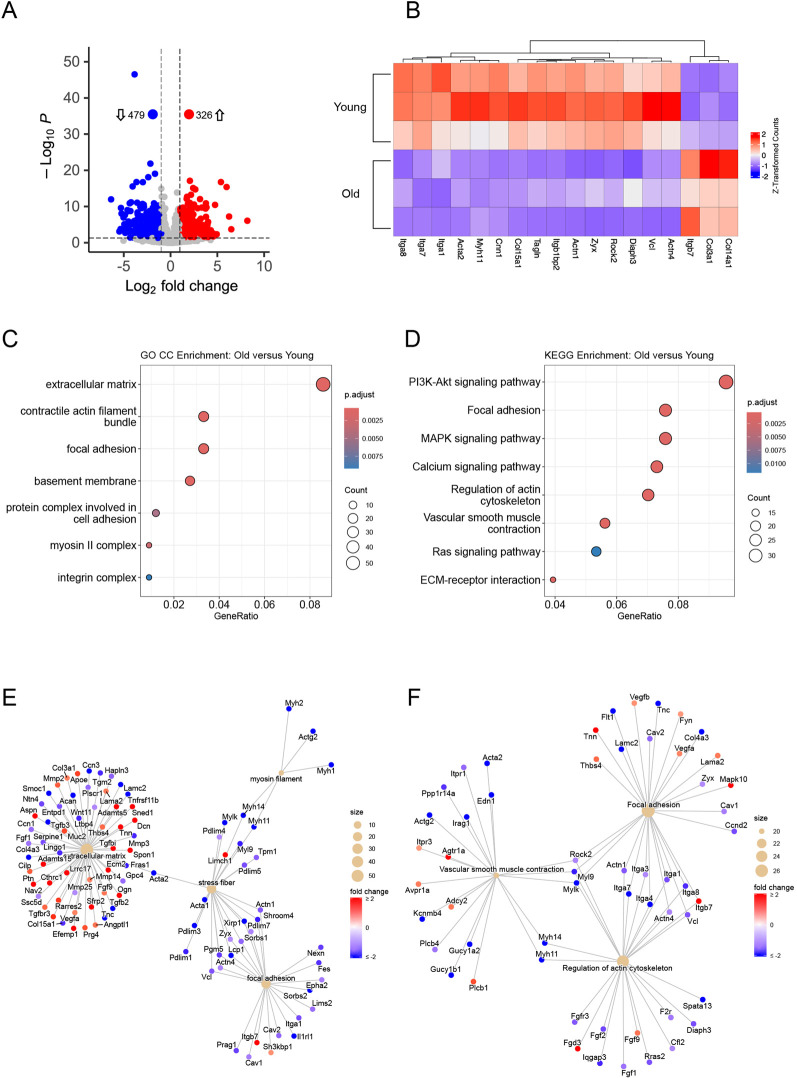
Differential gene expression analysis of aged versus young VSM cells. **(A)** Volcano plot showing total number of significant DEGs identified between the age groups. Red circles and blue circles indicate up and down regulated genes, respectively, old:young VSM cells. Grey circles indicate genes that failed significance thresholds. Dashed lines indicate significance thresholds at *p* < 0.05, and |log_2_ fold change| ≥ 1. **(B)** 2D heatmap showing the z-transformed counts for a subset of significant genes in old:young VSM cells. GO CC **(C)** and KEGG **(D)** term enrichment dot plots. Diameter of circles indicate the number of genes perturbed in the term sets, and the color gradient indicates the p-adjusted value for significance in the fold enrichment. GO CC **(E)** and KEGG **(F)** gene-pathway network plots. Beige circles indicate terms with the diameter reflecting the total number of genes perturbed. Genes are connected to terms based on known associations and are colored by the log_2_ fold change calculated during differential gene expression analysis. For visual presentation color shade does not change above 2 or below −2.

To identify genes of interest that were differentially regulated based on age, total RNA has been extracted, sequenced, and gene expression analyzed from both young and old VSM cells (n = 3 rats per each group). All comparisons were made with the young samples treated as the reference level against the aged samples. Differentially expressed genes (DEGs) were identified (Figure 1A) as having a p-adjusted value (*p*) < 0.05, and log_2_ fold change (fc) ≥ 1 or ≤ −1. A total of 326 upregulated and 479 downregulated DEGs (Figure 1A) were identified comparing the old:young VSM cells. The heatmap (Figure 1B) shows several genes involved in VSM actomyosin contractility and adhesion to the matrix, as well as genes encoding specific VSM marker proteins (Dinardo et al., 2014; Owens, 1995). These genes showed highly consistent expression patterns across biological replicates in each group. Aligning with our previous studies, we have shown by qRT-PCR that integrin β1 and several α-integrin subunits were downregulated in VSM cells isolated from old SFA. In addition, SMα-actin mRNA expression was also lower in VSM cells isolated from old SFA (Ojha et al., 2022). Moreover, previous fluorescence imaging showed that protein expression for vinculin and SMa-actin is also reduced in VSM cells isolated from old SFA (Seawright et al., 2018). At the same time, members of collagen family show upregulation in aging (Dinardo et al., 2014) as we also have shown in histological sections of aged SFA (Trache et al., 2020).

### 3.3 Vascular smooth muscle contractility pathway is age-dependent

Annotated term enrichment analysis was conducted on the 805 DEGs, to gain insight on the roles and pathways these genes have. Both the GO (Ashburner et al., 2000) and KEGG (Kanehisa et al., 2010; Kanehisa et al., 2017) *R. norvegicus* databases were queried using a *p* < 0.05, and fold enrichment >1 as thresholds to define enriched terms. The GO Cellular Component (GO CC) ontology (Figure 1C) revealed enrichment of cellular location/complex terms in the extracellular matrix (GO:0031012), contractile actin filament bundle (GO:0097517), focal adhesion (GO:0005925), basement membrane (GO:0005604), protein complex involved in cell adhesion (GO:0098636)/integrin complex (GO:0008305), and myosin II complex (GO:0016460). KEGG module enrichment (Figure 1D) similarly identified biological pathway enrichment in PI3K-Akt signaling (rno04151), focal adhesion (rno04510), MAPK signaling (rno04010), calcium signaling (rno04020), regulation of actin cytoskeleton (rno04810), vascular smooth muscle contraction (rno04270), Ras signaling (rno04014), and extracellular matrix-receptor interaction terms (rno04512). The pathway terms enriched here (Figures 1C,D) are consistent with previous studies in cannulated vessels highlighting the reduced contractility of aged SFA (Seawright et al., 2018; Seawright et al., 2016).

To further probe the gene level perturbation of the enriched terms, gene-pathway network plots (Figures 1E,F) were rendered to show interactivity of pathways with overlapping genes. In the GO CC enriched term network plot (Figure 1E), smooth muscle contractility genes such as Acta2, Myh11, and Vcl encoding vinculin protein that directly binds to actin showed strong association with cellular components related to contractile actomyosin bundle that regulates intracellular tension and focal adhesions, respectively. All these genes were significantly downregulated in aged vs. young VSM cells. Myosin filament, stress fiber, and focal adhesion/integrin complexes had most associated genes downregulated, indicating a significant reduction in aged VSM contractility compared to young. Extracellular matrix associated genes displayed a more dynamic response to aging, suggesting age-induced remodeling mechanisms that are associated with increased stiffness of the matrix (Kohn et al., 2015). The KEGG gene-pathway networks (Figure 1F) showed downregulation of several α and β integrins (Itga1/3/4/8, Itgb7) and vinculin that is associated with regulation of actin cytoskeleton and focal adhesion, while downregulation of Acta2 and Myh11 is associated to contractile function in VSM cells. Taken together, these results suggest that mechanisms related to cellular contractility and adhesion experience significant downregulation in aged vs. young SFA.

### 3.4 Short-duration pressure treatment restores VSM myogenic constriction in aged SFA

**FIGURE 2:**
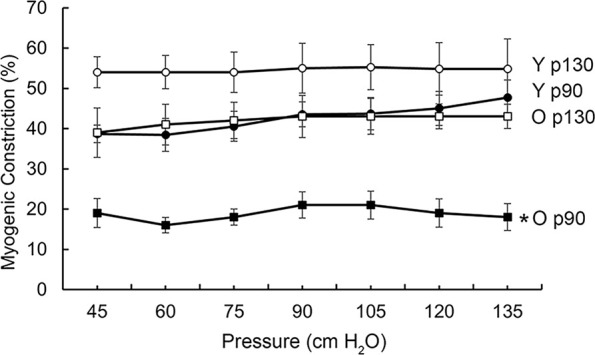
Effect of aging and pressure pre-treatment on myogenic constrictor responses in SFA. Myogenic responses were assessed in young and old SFA using 15 cm H_2_O step increases in pressure. Pre-treatment of old arteries with 130 cmH_2_O pressure for 1 h recovered myogenic response in old SFA. n = 4-5 rats/group. Data shown are mean ± SE. * Significance was evaluated at p < 0.05.

To evaluate the myogenic responses, SFA were exposed to increased pressures in a stepwise manner. Results showed that myogenic contractile responses were significantly lower in old SFA compared with young SFA at all pressures (Figure 2). Pre-treatment of old arteries with short-duration (1 h) high pressure (130 cm H_2_O) restored these constrictor responses in old arteries such that Old p130 was not different from Young p90. These data support our hypothesis that short-duration wall stretch improves myogenic contractility in aged SFA.

### 3.5 Cyclic stretch induces actin stress fiber formation in aged VSM cells

**FIGURE 3:**
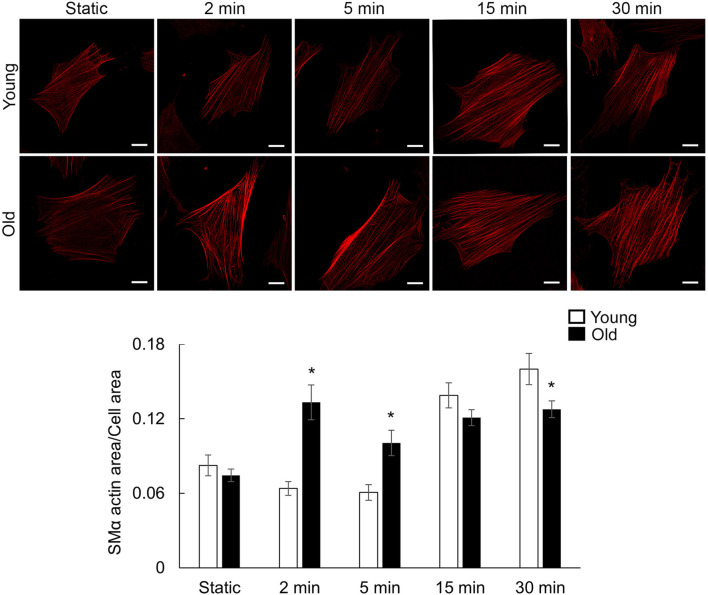
Representative confocal images of VSM cells plated on fibronectin-functionalized membranes. VSM cells have been exposed to cyclic stretch for different time duration 2, 5, 15, and 30 min and fluorescently labeled for SMα-actin. No stretch, static condition was used as control. Scale bar represents 10 μm. Quantitative fluorescence measurements are presented as mean ± SE. Significance was evaluated at p < 0.05. * Values are significantly different from young (n = 47–64 cells/group).

To evaluate the contribution of mechanical stimulation to VSM structural remodeling, we used an *in vitro* biaxial cyclic stretch system (Na et al., 2008) to mimic the circumferential stretch sensed by VSM cells in the vessel wall. Representative images of VSM cells plated on fibronectin functionalized membranes and immunofluorescently labeled for SMα-actin, a cytoskeletal protein essential for VSM contraction, show a progressive increase of actin fiber accumulation with increased duration of cyclic stretch, presenting more densely packed fibers along the long axis of the cell with time (Figure 3). Quantitative image analysis of fluorescence images (Figure 3; Supplementary Figure S1) showed that mechanical stretching increased smooth muscle α-actin (SMα-actin) protein area with old cells presenting more SMα-actin fibers at 2 min stretch which is maintained throughout the experimental time of 30 min. In contrast, SMα-actin protein area in young cells does not increase until 15 min stretch time, when they are not different from the old cells. These data show that stretch-induced mechanical stimulation is associated with an age- and time-dependent increase in SMα-actin fibers.

### 3.6 Cyclic stretch induces time-dependent protein recruitment at cell-matrix adhesions in aged VSM cells

**FIGURE 4:**
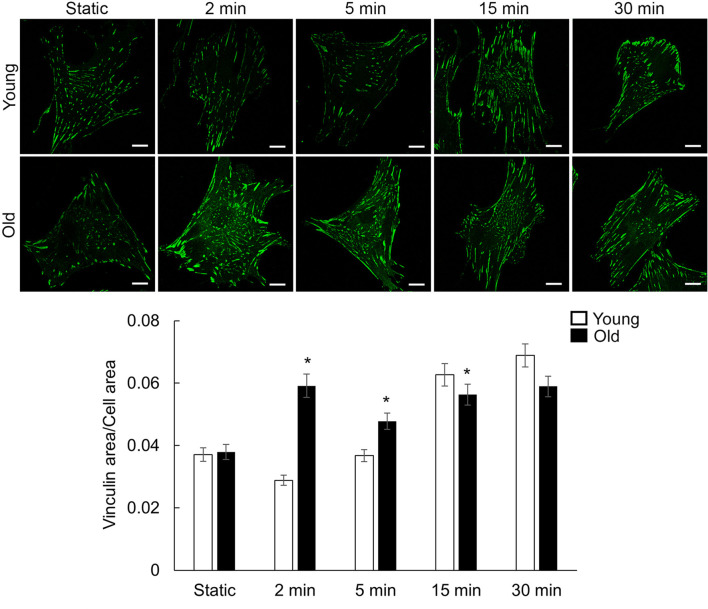
Representative confocal images of VSM cells plated on fibronectin-functionalized membranes. VSM cells have been exposed to cyclic stretch for different time duration 2, 5, 15, and 30 min and fluorescently labeled for vinculin. No stretch, static condition was used as control. Scale bar represents 10 μm. Quantitative fluorescence measurements are presented as mean ± SE. Significance was evaluated at p < 0.05. * Values are significantly different from young (n = 47–64 cells/group).

To further investigate how mechanical stretching affects VSM cell adhesion to the matrix, immunofluorescence staining was performed for both vinculin, as a cell-matrix adhesion marker, and integrin β1 involved in connecting the cell to the extracellular matrix substrate. When mechanical stretch is absent, there is no difference between young and old cells recruitment at cell-matrix adhesions for either vinculin or integrin β1 (i.e., integrin a5b1). Both cell groups show puncta-like small adhesions towards the center of the cell and an increase in adhesions towards the cell edges at higher stretch times. As the VSM cells were exposed to increased time of mechanical stretch, a similar time-dependent pattern of response was recorded for vinculin (Figure 4) as presented above for SMa-actin. Quantitative image analysis of fluorescence images (Figure 4; Supplementary Figure S2) showed that old cells responded immediately (2 min) to the external mechanical stretch by recruiting vinculin at cell-matrix adhesions but took a longer time (15 min) for the young cells to respond to the same exposure of the mechanical stretch.

**FIGURE 5:**
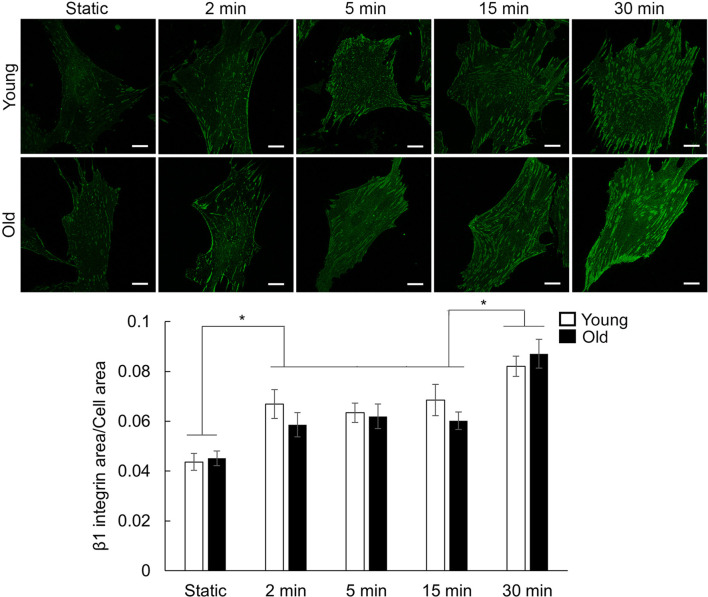
Representative confocal images of VSM cells plated on fibronectin-functionalized membranes. VSM cells have been exposed to cyclic stretch for different time duration 2, 5, 15, and 30 min and fluorescently labeled for integrin β1. No stretch, static condition was used as control. Scale bar represents 10 μm. Quantitative fluorescence measurements are presented as mean ± SE. Significance was evaluated at p < 0.05. * Values are significantly different between different stretch times (n = 40–61 cells/group).

Integrin β1 showed a different time-response pattern to the mechanical stretch than vinculin (Figure 5). Fluorescence image quantification showed no difference between the age groups at each stretch time point, however, a moderate integrin β1 recruitment increase for 2–15 min, followed by another step increase at 30 min was measured (Figure 5; Supplementary Figure S3). Taken together, these data show that stretch-induced mechanical stimulation induces significant time-dependent differential recruitment of vinculin and integrin β1.

### 3.7 Pressure treatment has a substrate-stiffness dependent effect on aged VSM cells

**FIGURE 6:**
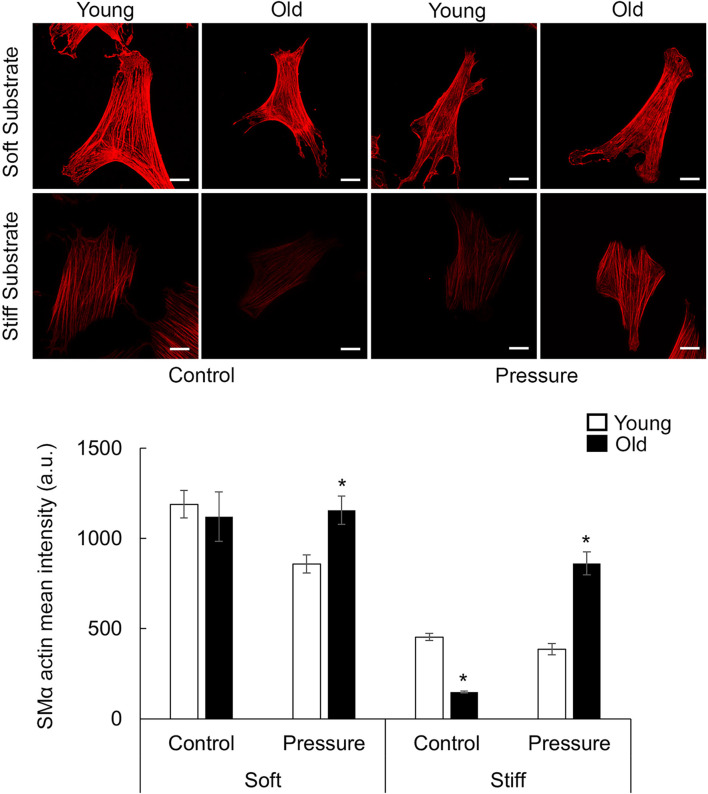
Representative confocal images of VSM cells plated on fibronectin-functionalized hydrogels of different stiffnesses (soft 4 kPa, stiff 100 kPa). VSM cells have been exposed to 16 kPa hydrostatic pressure for 30 min and fluorescently labeled for SMα-actin (n = 14–67cells/group). No pressure condition was used as control. Scale bar represents 20 μm. Quantitative measurements of overall fluorescence intensity are presented as mean ± SE. Significance was evaluated at p < 0.05. * Values are significantly different from young.

**FIGURE 7:**
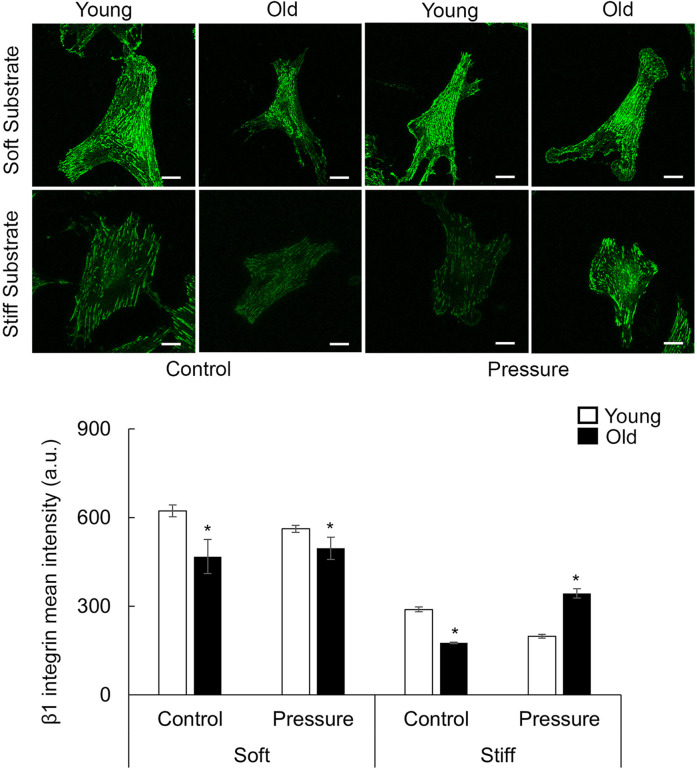
Representative confocal images of VSM cells plated on fibronectin-functionalized hydrogels of different stiffnesses (soft 4 kPa, stiff 100 kPa). VSM cells have been exposed to 16 kPa hydrostatic pressure for 30 min and fluorescently labeled for integrin β1 (n = 14-67 cells/group). No pressure condition was used as control. Scale bar represents 20 μm. Quantitative measurements of overall fluorescence intensity are presented as mean ± SE. Significance was evaluated at p < 0.05. * Values are significantly different from young.

Since the increase of intraluminal pressure has a significant effect in restoring myogenic contractility in aged SFA, we asked if the pressure treatment-induced changes may depend on the stiffness of the extracellular matrix. Thus, young and old VSM cells were cultured on soft and stiff substrates coated with fibronectin and subjected to 160 cm H_2_O pressure treatment (Swiatlowska et al., 2022). VSM cell morphology was assessed by immunofluorescence labeling for SMα-actin (Figure 6; Supplementary Figure S4) and integrin β1 (Figure 7; Supplementary Figure S5). While actin stress fibers and adhesions form on both substrate types, pressure treatment has no effect on aged cells on soft substrates, but there is a slight decrease in young cells with respect to control. Thus, the soft substrate reduces the age-dependent differences in mean fluorescence intensity of these proteins, and pressure has no effect on old cells. However, for cells plated on stiffer substates, SMα-actin and integrin β1 mean fluorescence intensities were significantly reduced in old cells compared with young control, while pressure treatment induced a significant recovery of the mean fluorescence intensity of these proteins in old but not young cells on stiff substrates.

**FIGURE 8:**
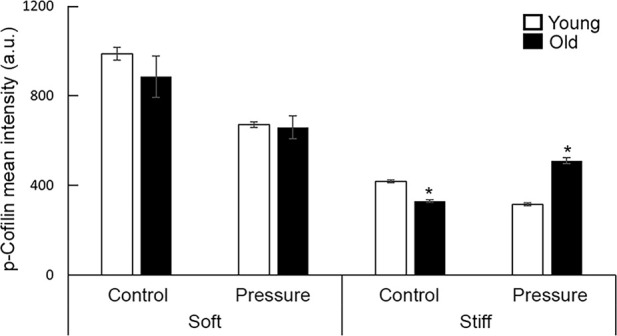
Quantitative measurements of overall fluorescence intensity VSM cells plated on fibronectin-functionalized hydrogels of different stiffnesses (soft 4 kPa, stiff 100 kPa). VSM cells have been exposed to 16 kPa hydrostatic pressure for 30 min and fluorescently labeled for p-cofilin (n = 14–67 cells/group). No pressure condition was used as control. Scale bar represents 20 μm. Measurements are presented as mean ± SE. Significance was evaluated at p < 0.05. * Values are significantly different from young.

Next, we asked whether pressure treatment-induced actin fiber formation is regulated by cofilin, a potent actin depolymerization factor (Kanellos et al., 2015). Quantification of mean fluorescence intensity of the inactive form of cofilin (i.e., phospho-cofilin) showed a good correlation between p-cofilin and SMα-actin upregulation in cells plated on stiff substrates (Figure 8; Supplementary Figure S6). For soft substrates, however, p-cofilin shows an age-independent slight decrease compared with control.

Taken together, these data suggest that the pressure treatment has a matrix stiffness-dependent beneficial effect in aging and is able to recover essential elements on the contractility pathway.

## 4 Discussion

Vascular aging is a physiological process characterized by progressive alteration of the structure and function of the arteries. Age-induced vascular remodeling is mostly characterized by arterial stiffening (Sehgel et al., 2015), which reduces the ability of large arteries to dampen the blood pressure wave as it travels to the microcirculation (O'Rourke and Safar, 2005). This, in turn, will transfer the pulsatile energy to the microvasculature leading to increased vascular resistance and impaired organ function (Maier et al., 2023). Pulse wave velocity measurements in conduit arteries are used clinically to determine vascular stiffness, but the importance of vascular stiffening in the microcirculation is not fully understood.

As aging progresses, there is a reduced expression of adhesion and contractile proteins within VSM cells which result in a phenotypic switch towards a more synthetic and hypertrophic state, eventually leading to elevated wall stiffness and reduced contractility of arteries (Seawright et al., 2018; Muller-Delp et al., 2018). The RNAseq analysis (Figure 1) confirmed at a larger scale previous findings related to a decrease in contractile and adhesion proteins in aged VSM cells. Both CC GO and KEGG pathway enrichment analysis showed that gene elements of actomyosin contractility pathway such as actin filament regulation, actin bundling, and myosin are downregulated in VSM cells isolated from SFA of aged Fischer 344 rats. Similar results were found for genes related to integrins and cell-matrix adhesion proteins (i.e., focal adhesions), while genes related to regulation and composition of extracellular matrix were partly upregulated. While our studies were able to identify key proteins of the matrix-integrin-actomyosin signaling pathway in aging, our understanding of how integrins (Martinez-Lemus et al., 2009) regulate actomyosin contractility and VSM stiffness and function in aging remains poorly understood (Borghesan and O'Loghlen, 2017).

Because exercise mitigates the effects of aging on VSM contractility, it has been proposed that short-duration stretch of resistance arteries due to increased arterial pressure during exercise, induces long-term changes in VSM phenotype and promotes healthy VSM function (Tanaka et al., 2000; Seals et al., 2008). Our previous studies showed that pressure treatment of resistance arteries *ex vivo* was able to recover agonist-induced vascular contractility (Seawright et al., 2016) and modulation of matrix stiffness regulates cytoskeletal architecture improving age-dependent reduction of actin fiber formation (Ojha et al., 2022). Therefore, in the current study we used a biophysical approach to assess the effects of mechanical stimulation induced by the increase in intraluminal pressure in the artery. We hypothesized that age-induced impairment of VSM contractility can be rescued by mechanical stimulation that enhances formation of SMα-actin fibers and cell-matrix adhesions in aged VSM cells. Our results showed that: (i) preconditioning of old arteries with short-duration high pressure treatment restored the myogenic constrictor responses in old arteries; (ii) stretch-induced mechanical stimulation is associated with an age- and time-dependent increase in SMα-actin fibers and adhesion proteins; and (iii) pressure treatment has a matrix stiffness-dependent beneficial effect in aging by recovering essential elements on the contractility pathway.

The decrease of myogenic reactivity with age may contribute to abnormal local blood flow control and diminished orthostatic tolerance in aging (Delp and Armstrong, 1988). Results from functional experiments performed on *ex vivo* canulated SFA, showed that pre-conditioning of old SFA with a short-duration high pressure treatment within a range of pressure experienced during a bout of exercise (Williams and Segal, 1993) restored the myogenic constrictor responses in old arteries, however, the pressure treatment had little effect on SFA isolated from young rats (Figure 2). The beneficial effect of pressure treatment applied here is in good agreement with our previous measurements showing improvements of the age-induced impairments of agonist-induced VSM contractility (Seawright et al., 2016). Although present results revealed that pressure treatment improved contractility in old SFA *ex vivo*, future studies will be needed to determine how long the improved function persists and whether repeated exposures to pressure, as would occur with exercise training, induce a greater improvement in function than a single exposure.

Regulation of local skeletal muscle blood flow is mediated in large part by alterations in diameter of resistance arteries in response to sympathetic neurotransmitter norepinephrine/noradrenaline and changes in intraluminal pressure activating myogenic reactivity. There have been differential effects of aging on responsiveness of rat resistance arteries in several muscle types to norepinephrine with unaltered constriction of arterioles from cremaster, gastrocnemius and soleus muscles (Muller-Delp et al., 2002; Cook et al., 1992), and reduced constriction of feed arteries from aged soleus muscle (Seawright et al., 2016; Seawright et al., 2018). One study reported enhanced constriction of soleus muscle arterioles to norepinephrine (Donato et al., 2007), but vessel reactivity was assessed only in the presence of β-blocker propranolol, which reduced β-mediated vasodilation. Based on their earlier report of unaltered soleus arteriolar constriction with aging in the absence of propranolol (Muller-Delp et al., 2002), the authors concluded that smooth muscle contractility of these arterioles in response to norepinephrine was unaffected by age. By contrast, myogenic constriction is reduced in rat arterioles from both gastrocnemius and soleus muscle (Muller-Delp et al., 2002), as well as SFA. Blood flow regulation by SFA has been suggested to rely more on myogenic constriction than on adrenergic effects (Delp and Armstrong, 1988), with myogenic responses impacting blood flow when mean arterial pressure is altered during changes in activity or postural/orthostatic challenges (Lipsitz, 1985).

Circumferential wall stretch arises from the radial expansion of the vessel wall as intraluminal pressure increases (Trache et al., 2020). Beyond the pressure-induced tension exerted on the extracellular matrix, cyclic stretch is involved in regulating the homeostasis of VSM cells by enhancing protein expression (Dinardo et al., 2014; Song et al., 2012). Our results showed that cyclic stretch induced faster formation of actin stress fibers and recruitment of vinculin at cell-matrix adhesion in old cells than young cells and sustained this effect throughout the experimental time (Figures 3, 4). However, integrin β1 showed a different time-response pattern to the mechanical stretch than vinculin (Figure 5) with a time-dependent step increase in its recruitment to cell-matrix adhesions. The differential time recruitment to cell-matrix adhesions for vinculin and integrin β1 may be due to the stability of each protein at adhesions that relates with their different turnover time, about 2 min for vinculin (Humphries et al., 2007) and more than 6 min for integrins (Ballestrem et al., 2001). These data suggest that stretch-induced mechanical stimulation is associated with an age- and time-dependent active remodeling of the VSM cell cytoskeletal architecture needed to keep cellular attachment and adapt to the applied stretch.

Several studies suggested that calcium-mediated MLCK activation/myosin phosphorylation are not the only mechanisms involved in myogenic response (D’Angelo et al., 1997; Osol et al., 2002). The pressure-dependent effects on VSM cells most likely involve a pressure-sensor at the membrane level that is activated by changes in the intraluminal pressure (Davis 2012). Thus, the remodeling of integrin-based adhesions is an important factor in the recovery of VSM cells contractility by exposure to mechanical stimulation (Kuo, 2013). Integrins are uniquely positioned to provide bi-directional signaling at VSM cell membrane. Integrins can sense pressure-induced changes in the extracellular matrix and trigger intracellular biochemical signaling to activate actomyosin contractility required for development of myogenic constriction (Hill and Meininger 2012). Our studies showed that pressure treatment induced a significant recovery of integrin β1 and SMα-actin expression in old but not young cells on stiff hydrogels (Figures 6, 7). Increased formation of SMα-actin fibers was also supported by an increase in the inactive form of p-cofilin (Figure 8). Since aged VSM cells experience stiffer extracellular matrix environment *in vivo* in old arteries, we propose that pressure-preconditioning of old SFA *ex vivo* provides means for activation of mechanotransduction pathways, altering integrin dynamics and increasing VSM cell contractility, further facilitating improvement of myogenic response measured in pressure-preconditioned old SFA.

**FIGURE 9:**
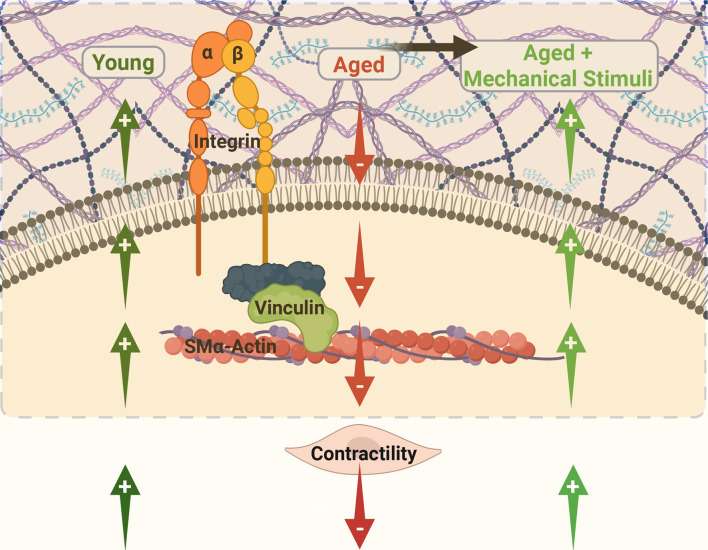
Integrin α5β1 is known to be involved in regulating cellular and vessel contractility. In young cells, actomyosin apparatus is strong and the VSM cell is able to properly regulate its stiffness and adhesion to the matrix in response to mechanical signals. In aging, the decrease in SMα-actin stress fiber formation and alteration of cell-matrix adhesions induces a reduced VSM cell contractility and deficient mechanosensing. However, mechanical signals (e.g., stretch, pressure) presented to aged VSM cells through the extracellular matrix stimulate integrin activity which in turn promotes enhanced actomyosin-induced contractility, thus, recovering the age-induced effects. Diagram created with www.biorender.com.

In summary, the results of this study suggest that extracellular mechanical cues are able to restore integrin-dependent contractility in VSM cells isolated from aged resistance arteries (Figure 9). Our data show that discrete VSM cell mechanical properties (cell stiffness and adhesion) and their ability to adapt to external mechanical signals (i.e., intraluminal pressure) are important contributors to regulation of vascular contractility in aging.

## Data Availability

The data presented in the study are deposited in the Gene Expression Omnibus repository, accession number GSE297824, and figshare repository, https://doi.org/10.6084/m9.figshare.29214467.

## Supplementary material

SUPPLEMENTARY FIGURE S1
Quantitative measurements of SMα-actin fluorescence from Figure 3 are presented as box and whisker plot. Mean value shown by ‘x’. All data points are represented. Significance was evaluated at p < 0.05.

SUPPLEMENTARY FIGURE S2
 Quantitative measurements of vinculin fluorescence from Figure 4 are presented as box and whisker plot. Mean value shown by ‘x’. All data points are represented. Significance was evaluated at p < 0.05.

SUPPLEMENTARY FIGURE S3
Quantitative measurements of integrin β1 fluorescence from Figure 5 are presented as box and whisker plot. Mean value shown by ‘x’. All data points are represented. Significance was evaluated at p < 0.05.

SUPPLEMENTARY FIGURE S4
Quantitative measurements of overall fluorescence intensity of SMα-actin from Figure 6 are presented as box and whisker plot. Mean value shown by ‘x’. All data points are represented. Significance was evaluated at p < 0.05.

SUPPLEMENTARY FIGURE S5
Quantitative measurements of overall fluorescence intensity of integrin β1 from Figure 7 are presented as box and whisker plot. Mean value shown by ‘x’. All data points are represented. Significance was evaluated at p < 0.05.

SUPPLEMENTARY FIGURE S6
Quantitative measurements of overall fluorescence intensity of p-cofilin from Figure 8 are presented as box and whisker plot. Mean value shown by ‘x’. All data points are represented. Significance was evaluated at p < 0.05.
