# Supplementary figures and images for: Mechanical signaling regulates vascular smooth muscle cell adaptation in aging
Source: Front Physiol. 2025 Jul 14;16:1593886. doi: 10.3389/fphys.2025.1593886 (PMC12301766; doi:10.3389/fphys.2025.1593886)

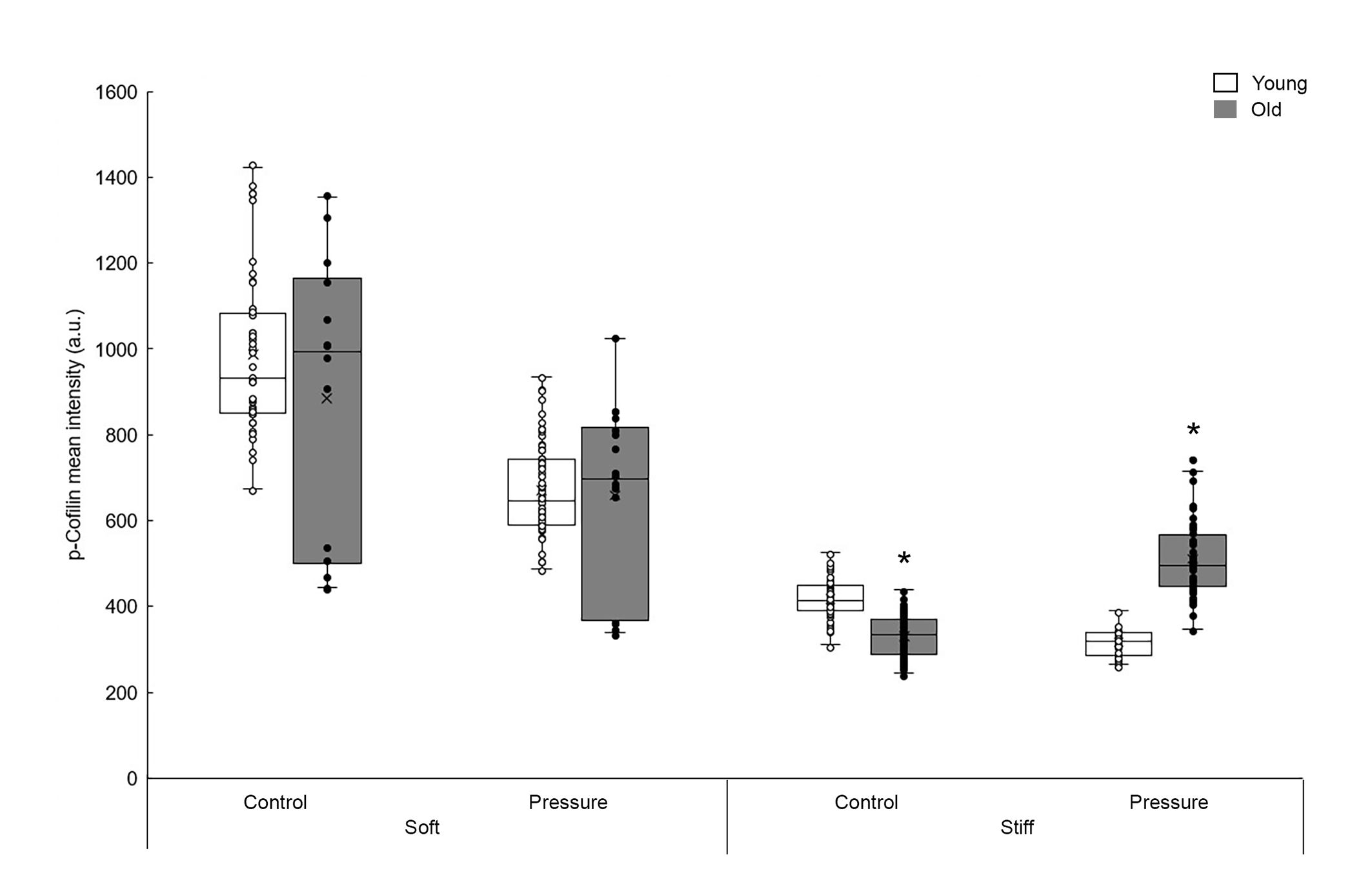

Supplement: Supplementary file 1 [file Image6.tif]

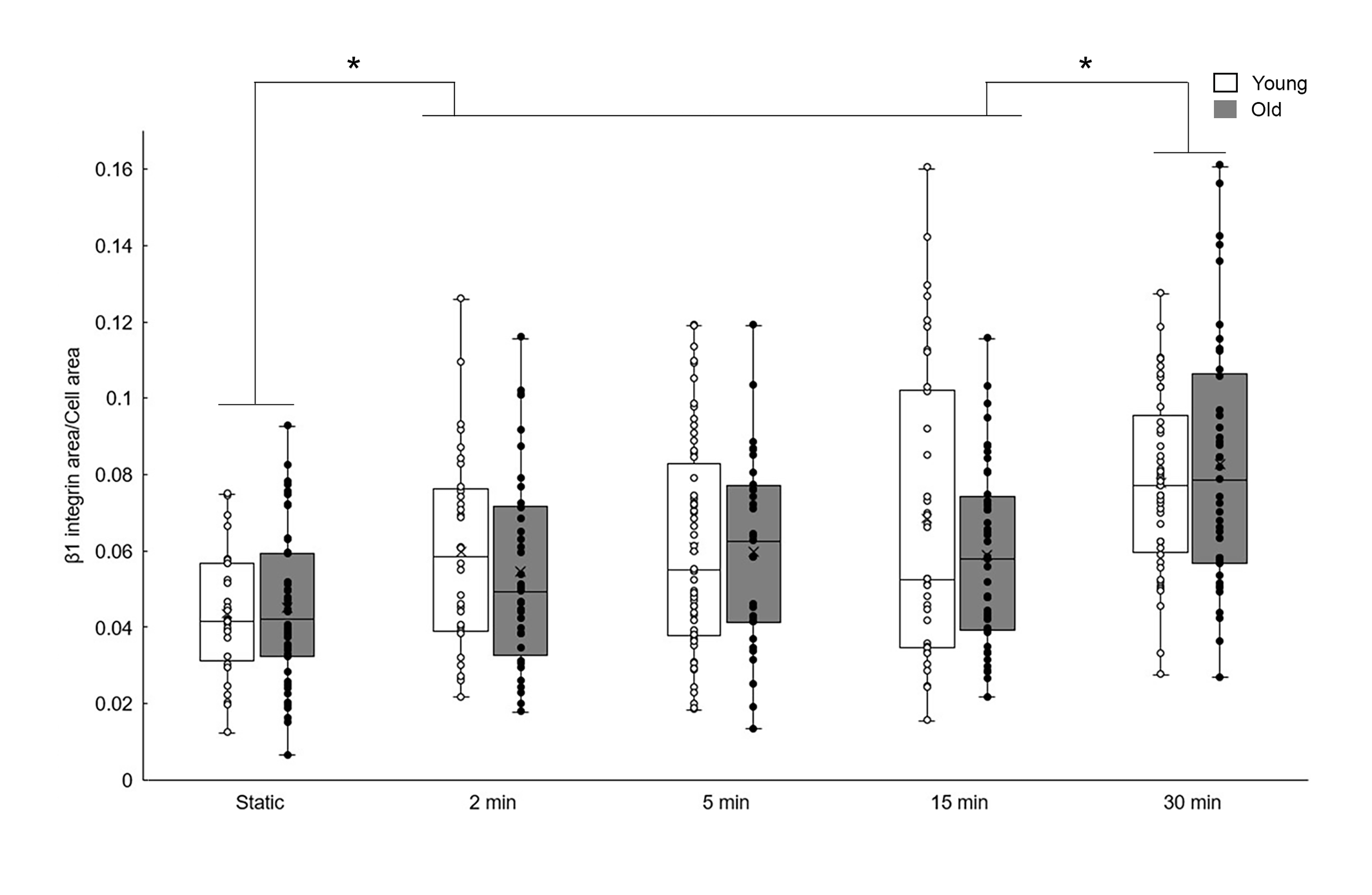

Supplement: Supplementary file 2 [file Image3.tif]

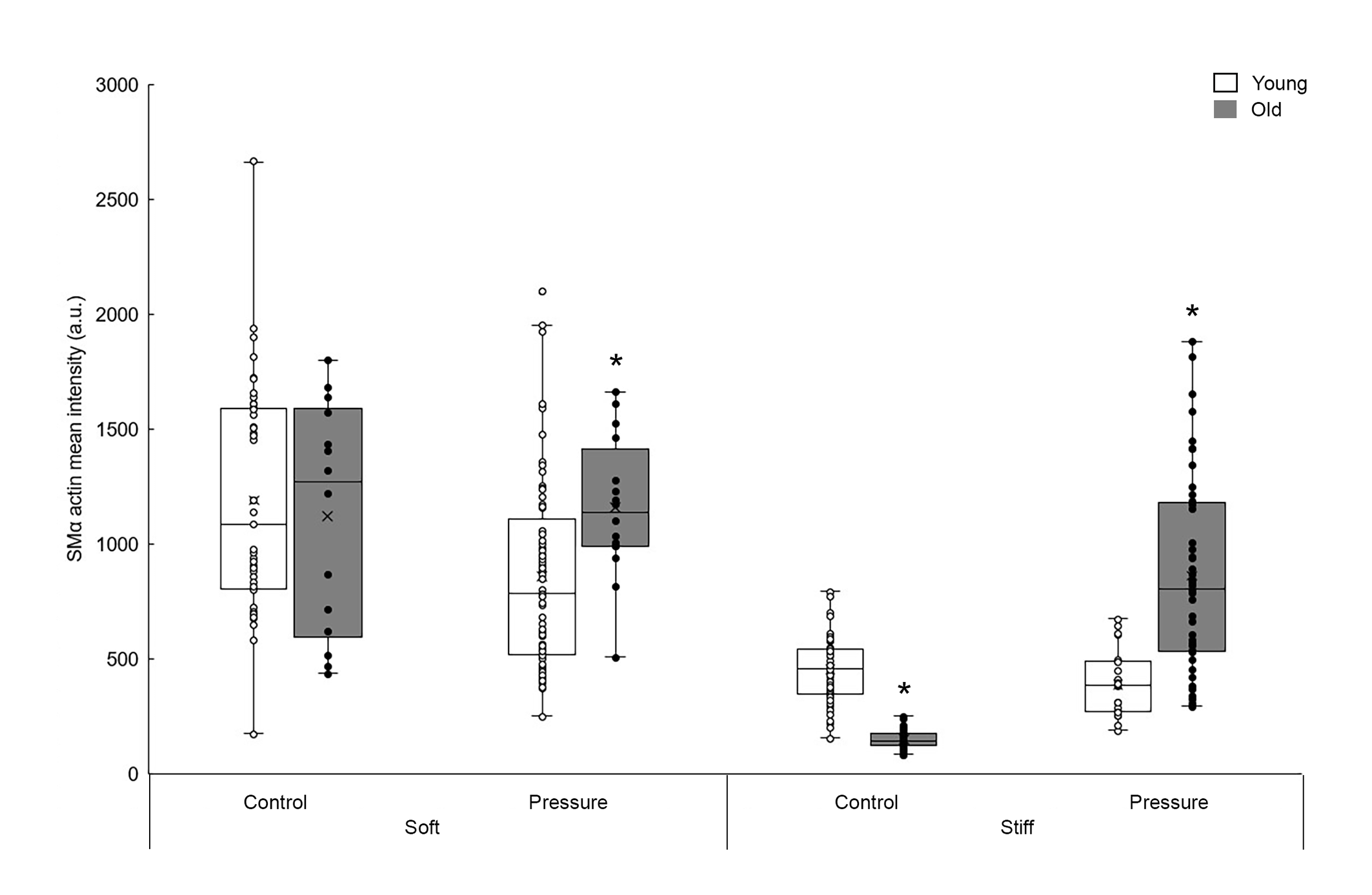

Supplement: Supplementary file 3 [file Image4.tif]

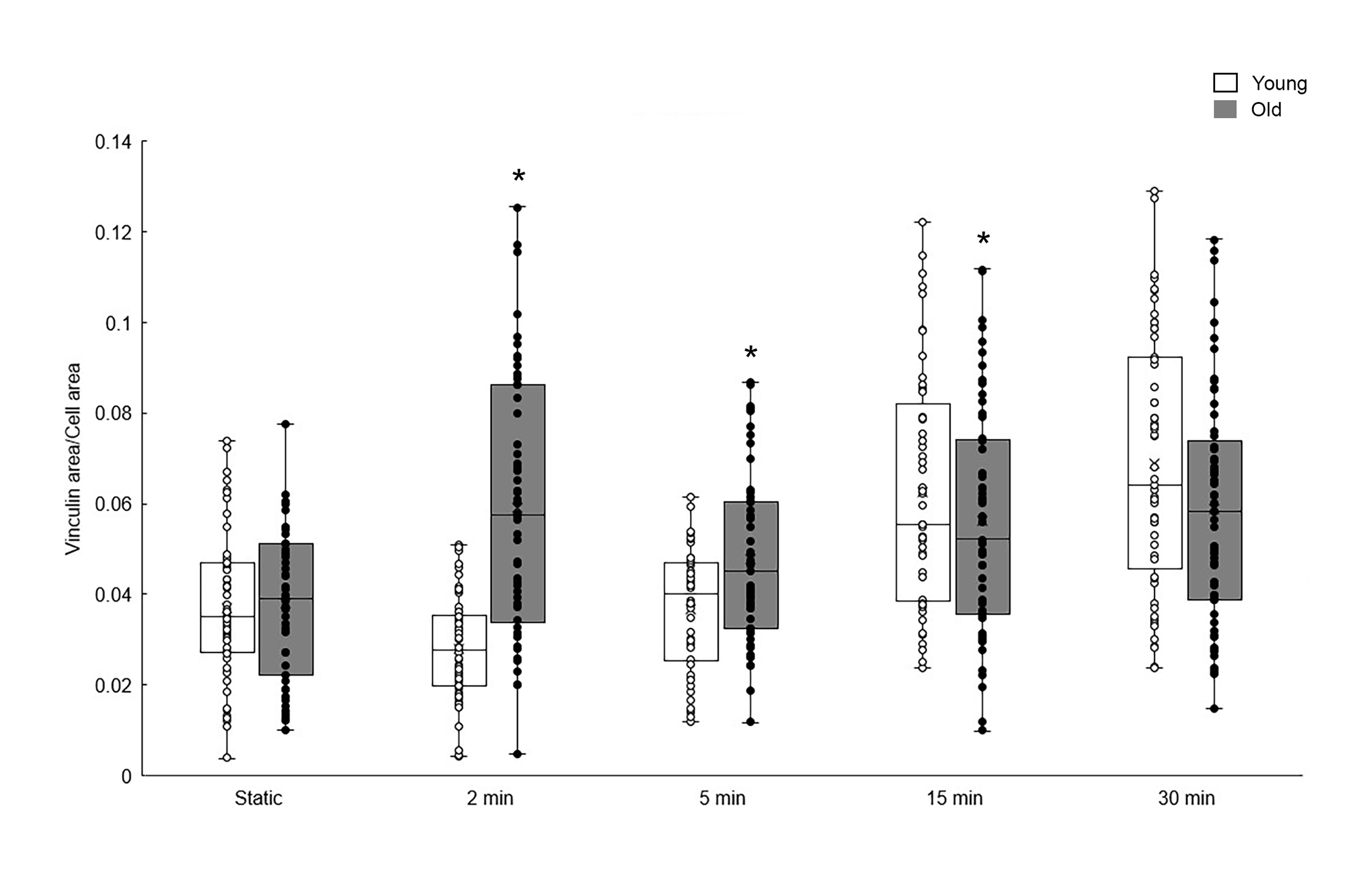

Supplement: Supplementary file 4 [file Image2.tif]

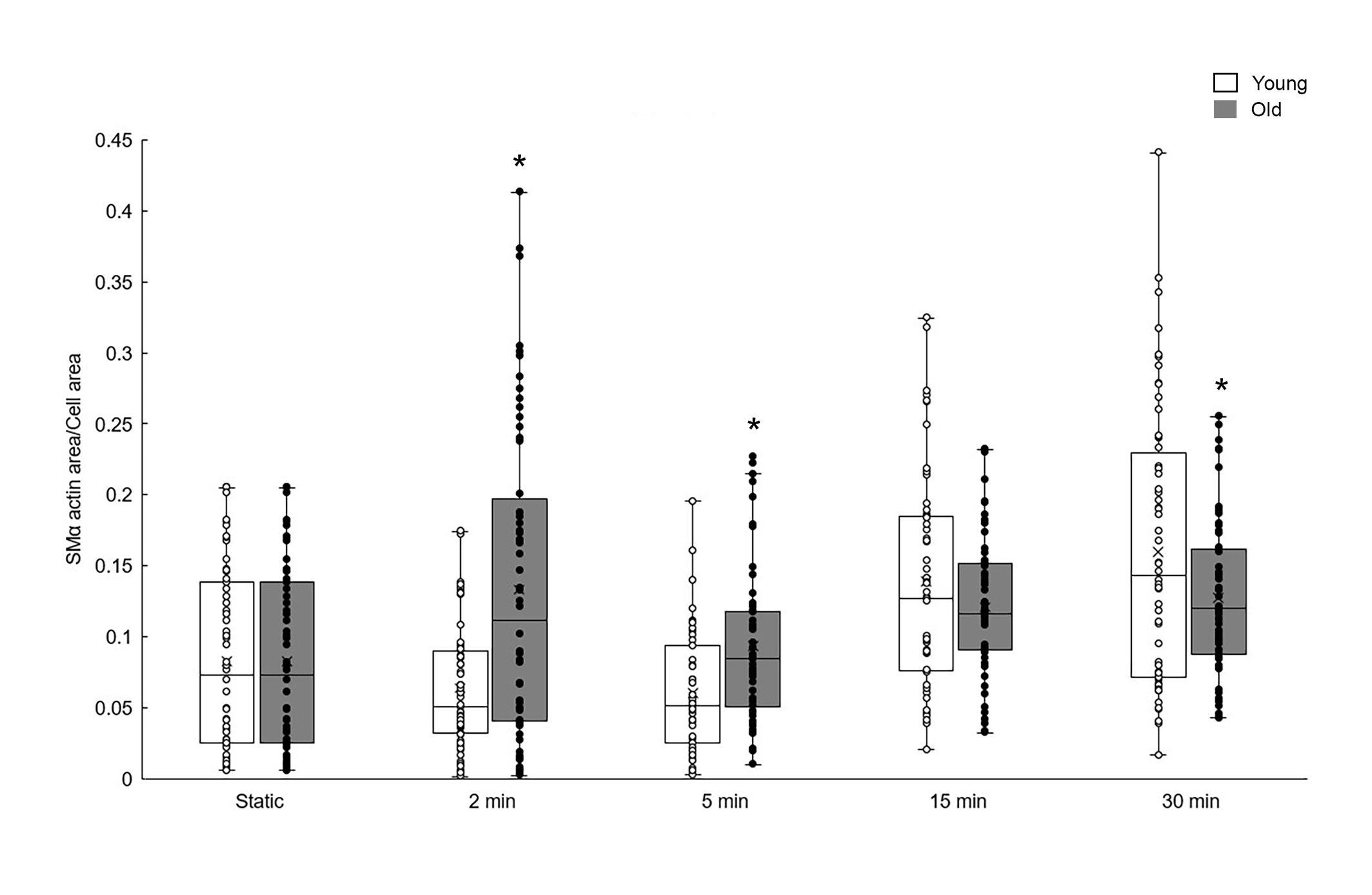

Supplement: Supplementary file 5 [file Image1.tif]

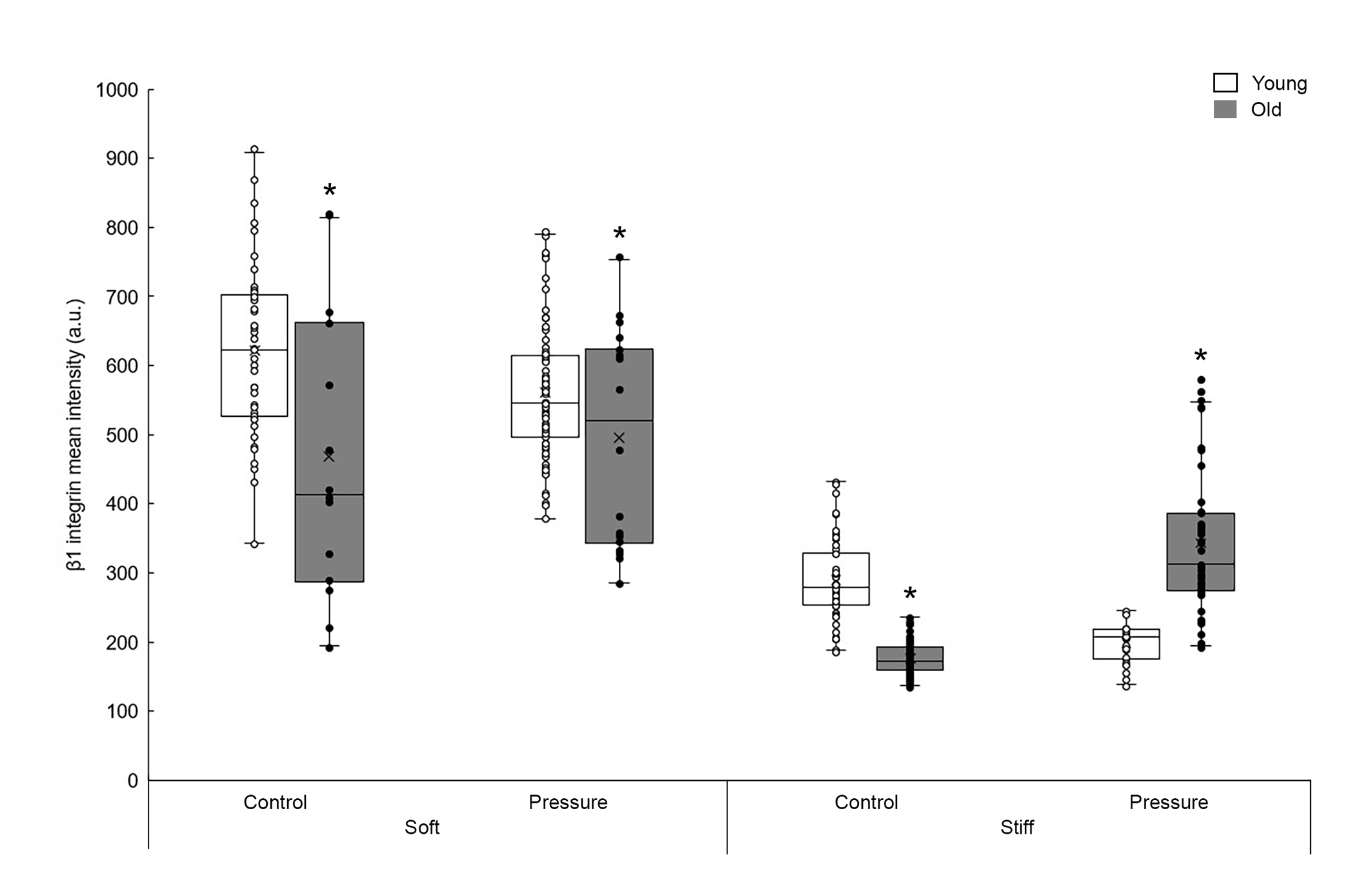

Supplement: Supplementary file 6 [file Image5.tif]
